# Purification and Identification of Novel Antioxidant Peptides from Enzymatically Hydrolysed *Samia ricini* Pupae

**DOI:** 10.3390/molecules26092588

**Published:** 2021-04-29

**Authors:** Nattakarn Wongsrangsap, Suttida Chukiatsiri

**Affiliations:** Department of Biochemistry, Faculty of Science, Kasetsart University, 50 Ngam Wong Wan Road, Ladyao, Chatuchak, Bangkok 10900, Thailand; nattakarn.won@ku.th

**Keywords:** *Samia ricini* pupae, enzymatic protein hydrolysates, antioxidant peptides, antioxidant activity

## Abstract

The emergence of excessive free radicals leads to the destruction of various systems within the body. These free radicals also affect nutritional values, color, taste, and emit an odor akin to rancid food. Most food industries use synthetic antioxidants, such as BHT (butylated hydroxytoluene) or BHA (butylated hydroxy anisole). However, high doses of these can be harmful to our health. Therefore, an antioxidant compounds, such as bioactive peptides from edible animals or plants, have emerged to be a very promising alternative as they reduce potential side effects. This study focused on the purification and identification of antioxidant peptides from protein hydrolysates of wild silkworm pupae (*Samia ricini*). Antioxidant peptides were purified from the hydrolysate by ultrafiltration and RP-HPLC. The results showed that protein hydrolysate from *S. ricini* pupae by trypsin with a molecular weight lower than 3 kDa and highly hydrophobic property, exhibited strong DPPH radical scavenging activity and chelating activity. Further identification of peptides from the fraction with the highest antioxidant activity was carried out using LC-MS/MS. Three novel peptides, i.e., Met-Ley-Ile-Ile-Ile-Met-Arg, Leu-Asn-Lys-Asp-Leu-Met-Arg, and Glu-Asn-Ile-Ile-Leu-Phe-Arg, were identified. The results of this study indicated that the protein hydrolysate from *S. ricini* pupae possessed potent biological activity, and the novel antioxidant peptides could be utilized to develop health-related antioxidants in food industry.

## 1. Introduction

Free radicals are derived either from normal essential metabolic processes in the human body or from external sources. Many radicals are highly reactive. They play major roles in biochemical pathways and food degradation [1,2,3]. These also affect color, taste, odor, and nutritional values of food [4,5]. BHT (butylated hydroxytoluene) and BHA (butylated hydroxy anisole) are two widely used synthetic antioxidants in foods. However, they display some toxic and hazardous effects on human health [6,7]. These effects include attention-deficit hyperactivity disorder (ADHD), allergy, and dermatitis, or contribute to cancer [8,9]. Thus, more studies have focused on natural antioxidants.

*Samia ricini* is a non-mulberry, multivoltine, domestic sericigenous insect, largely reared by the farmers of northeastern states of India, where it is grown primarily for silk production and food. *S. ricini* pupae are by-products of silk industry. Previous showed that *S. ricini* pupae contain significantly high levels of phenolic [10]. According to the protein digestibility corrected amino acid score (PDCAAS), *S. ricini* pupae achieved a score of 87%. They also have 54% protein contents of dry weight, with abundant essential amino acids, such as methionine, leucine, phenylalanine, lysine, valine, and isoleucine [11]. *S. ricini* pupae contain essential fatty acids, such as α-linolenic acid, arachidic, lignoceric, and important vitamins and minerals, such as phosphorus, iron, calcium, and magnesium [12]. In addition, protein extracts from *S. ricini* pupae have been identified to exert anticancer effects by downregulating the expression of IL-6, IL-1β and TNF-α through biomolecular changes in human breast cancer cells [13]. However, antioxidant peptides from *S. ricini* pupae have not been identified.

In this study, three novel antioxidant peptides from enzymatically hydrolyzed *S. ricini* pupae were purified and identified. Protein extracts from *S. ricini* pupae were hydrolyzed by trypsin and pepsin. Antioxidant activities were evaluated with DPPH and iron (FeII) chelation. The antioxidant peptides were purified by reverse-phase high performance liquid chromatography (RP-HPLC), and the amino acids sequences were identified by liquid chromatography with tandem mass spectrometry (LC-MS/MS).

## 2. Materials and Methods

### 2.1. Materials and Reagents

*Samia ricini* pupae were provided by the Kasetsart University Smart Silk Centre, Kasetsart University, Thailand. Chemicals and reagents were purchased from the following organizations: Sodium chloride (NaCl), ethylenediaminetetraacetic acid (EDTA), and sodium dodecyl sulfate (SDS)—from Bio Basic Inc. (Markham, ON, Canada), TritonX-100—from Scharlau Chemie S.A. Co. (Barcelona, Spain), phenylmethylsulfonyl fluoride (PMSF) and hydrochloric acid (HCl)—from Sigma-Aldrich, Inc. (Munich, Germany), Pepsin from ELITechGroup Inc. (Washington, WA, USA), 1,10-phenanthroline monohydrate—from Univar Solutions Inc. (Denver, CO, USA), acetonitrile—from VWR International LLC. (Fontenay-sous-Bois, France), trifluoroacetic acid (TFA)—from Thermo Fisher Scientific Inc. (Leics., UK). All other reagents used were of analytical grade and were purchased from Sigma-Aldrich Inc. (St. Louis, MO, USA), or were available commercially.

### 2.2. Preparation of Protein Extraction S. ricini

*S. ricini* was weighed to blend with a buffer solution consisting of 50 mM Tris-HCl (pH 7.4), 150 mM NaCl, 1 mM EDTA, 1% TritonX100, 0.1% SDS and 4 mM PMSF, then sampled by centrifuge at 14,000× *g* at 4 °C for 10 min. The supernatant obtained was dialyzed overnight (O/N) with distilled water which was changed at least three times, every 4 h crude protein concentrations were measured using a nanodrop microvolume UV–VIS spectrophotometers (Thermo Fisher Scientific Inc., Waltham, MA, USA).

### 2.3. Preparation of Hydrolysates and Fractionation with Ultrafiltration Membranes

The sample was dissolved in distilled water at a concentration of 25 mg/mL and hydrolyzed for 12 h using trypsin at pH 8.0 at 37 °C and papain at pH 6.0 at 50 °C for 6 h using pepsin at pH 2.2 at 37 °C (1 mg of enzyme/25 mg protein), followed by enzyme activity termination by heating for 10 min in boiling water. The samples were then centrifuged at 9000× *g* at 4 °C for 15 min, and the supernatants stored at −20 °C for further analysis.

### 2.4. Purification of the Antioxidant Peptides from S. ricini Pupae

#### 2.4.1. Ultrafiltration

The hydrolysate sample was fractionated using ultrafiltration membrane (Millipore, Darmstadt, Germany) according to the instructions in the manual (Amicon^®^ Ultra filter) with MW cut-offs of 3, 10, and 30 kDa. The permeates or retentates were collected as >30 kDa, <30 kDa, 10–30 kDa, <10 kDa, 3–10 kDa, and <3 kDa, respectively. All permeates were stored at −20 °C for further analysis.

#### 2.4.2. RT-HPLC

*S. ricini*, hydrolyzed with trypsin, was purified by RT-HPLC (Perkin Elmer 200 Series, PerkinElmer Inc., Waltham, MA, USA) according to the method of Xing, L.-j., et al. [14] with a slight modification. Purification was carried out using a Thermo C18 column (diameter 4.5 mm, length 250 mm, particle size 5 µm, Thermo Fisher Scientific Inc., Waltham, MA, USA). For separation part, solvent composition of the mobile phase at the two channels was 0.1% (*v/v*) formic acid and 2% (*v/v*) acetonitrile for channel A and 0.1% (*v/v*) formic acid and 100% (*v/v*) acetonitrile for channel B. The peptides were separated by a gradient elution to 30% B in 2 min, to 80% B in 18 min, to 100% B in 7 min.

The flow rate was set at 0.3 mL/min, measured at wavelengths of 220 nm, and collected fractions were stored at −20 °C for further analysis.

#### 2.4.3. LC-MS/MS

*S. ricini* fractions were determined with a 6420 triple quadrupole LC/MS (Agilent Technologies, Inc., Santa Clara, CA, USA), coupled with an electrospray ionization source (ESI), recording the mass spectral range of 50–1506 *m/z*. The linear gradients of solution A and solution B were based on the RP-HPLC method.

### 2.5. Determination of Antioxidant Activity

#### 2.5.1. DPPH Radical Scavenging Activity

DPPH radical scavenging activity was measured according to the method of Blois, M. S. J. N. [15]. The sample was diluted with deionized water (BHA and BHT were used as positive controls) and added to 50 µL of 0.125 mM 2,2-diphenyl-1-picrythyhydrazyl radical (DPPH) in 70% ethanol. The mixture was left at room temperature for 30 min in the dark, and the absorbance was measured at 517 nm (NanoDrop ONE, Thermo Fisher Scientific Inc., USA). The activity was expressed as a percentage DPPH scavenging relative to control, using the following equation:

% Radical scavenging activity = (1 − (*A_sample_*/*A_control_*)) × 100

where *A_sample_* is the absorbance of sample and *A_control_* is the absorbance of the control. The IC_50_ value is defined as an inhibitory concentration of sample that is required to scavenge 50% of radical activity.

#### 2.5.2. Iron Chelating Activity

The iron chelating activity was measured according to the method of Chaudhary, S., et al. [16]. Sample and control were prepared, and were added to 30 µL of methanol and 20 µL of 1, 10-phenanthroline-iron (III). 1, 10-henanthroline-iron (III) was prepared by 0.198 g of 1, 10-phenanthroline monohydrate, 2 mL of 1 M hydrochloric acid, and 0.16 g mixed to incubated at 50 °C for 30 min in the dark, and the absorbance was measured at 510 nm (NanoDrop ONE, Thermo Fisher Scientific Inc., USA). The activity was expressed as a percentage of iron chelating activity to control, using the following equation:

% Chelating activity = (1 − (*A_sample_*/*A_control_*)) × 100

where *A_sample_* is the absorbance of sample and *A_control_* is the absorbance of the control. The IC_50_ value is defined as an inhibitory concentration of sample that is required to chelate 50% of activity.

### 2.6. Statistical Analysis

The experiments were performed in triplicate (n = 3) and the results expressed as the mean ± standard deviation (SD) by GraphPad Prism 8.0.1 (244) (GraphPad Software, San Diego, CA, USA). One-way analysis of ANOVA (and nonparametric or mixed) indicated significant differences.

## 3. Results

### 3.1. Preparation of Protein Hydrolysates of S. ricini Pupae

In this research, different proteolytic enzymes were used, including trypsin and pepsin. DPPH is a commonly-used method of assessing antioxidant abilities as it is a simple, easy, inexpensive, and precise method, and the best method in in vitro [17,18,19,20,21]. Iron chelating is a test of iron-binding capacity, as an excess of iron in the body can also lead to an increase of free radicals [22,23]. The following Table 1 and Table 2 show the antioxidant activity of protein hydrolysates with trypsin and pepsin, comparable with BHA (positive control). The DPPH scavenging activity presented as IC_50_ values is shown in Table 1. Protein hydrolysate with trypsin (IC_50_ = 1406.6 ± 86.9 µg/mL), and with pepsin (IC_50_ = 1495.6 ± 101.7 µg/mL) showed no significant difference when compared with BHA (IC_50_ = 1304.5 ± 75.4 µg/mL). However, protein hydrolysate with pepsin exhibited the strongest chelating activity (IC_50_ = 189.3 ± 57.1 µg/mL) compared to protein hydrolysate with trypsin (IC_50_ = 286.5 ± 48.7 µg/mL), or BHA (IC_50_ = 297.8 ± 29.6 µg/mL) (Table 2).

### 3.2. Purification of Antioxidant Peptides from S. ricini Pupae

#### 3.2.1. Ultrafiltration

**Figure 1:**
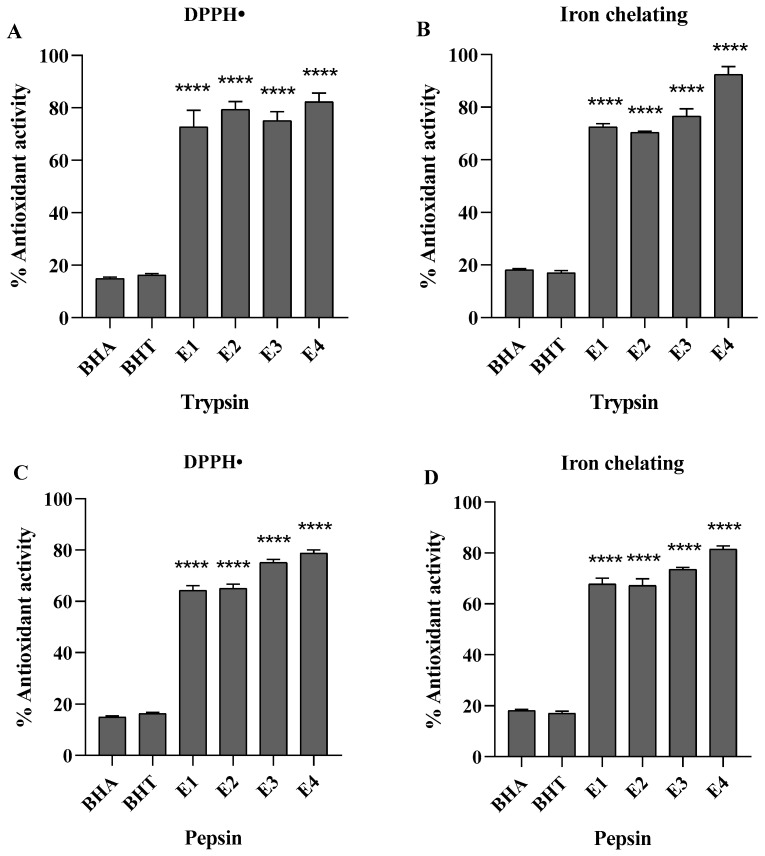
DPPH radical scavenging and iron-chelating activities of fractions from ultrafiltration. (**A**) DPPH radical scavenging activity of fractions from protein hydrolyzed with trypsin. (**B**) Iron-chelating activity of fractions from protein hydrolyzed with trypsin. (**C**) DPPH radical scavenging activity of fractions from protein hydrolyzed with pepsin. (**D**) Iron-chelating activity of fractions from protein hydrolyzed with pepsin. All samples were tested at concentration of 1.0 mg/mL. Data are presented as mean ± standard deviation (SD) (n = 3, **** *p* < 0.0001, unpaired Student *t*-test).

The resulting protein hydrolysates are the acquisition of a crude peptide consisting of products of side reaction and a mixture of peptide. Ultrafiltration membranes are used for purification of biological compounds [24]. Protein hydrolysates of *S. ricini* pupae were fractionated by ultrafiltration using three molecular weight cut-off (MWCO) membranes (3, 10, 30 kDa), and four fractions, namely, E1 (MW > 30 kDa), E2 (10 kDa < MW < 30 kDa), E3 (3 kDa < MW < 10 kDa) and E4 (MW < 3 kDa) were collected. Antioxidant activities of protein hydrolysate with trypsin, comparable with BHA and BHT (positive control), are shown in Figure 1A,B. The results showed that protein hydrolysate contributes effectively to scavenging free radicals, and exhibited significant antioxidant activity compared to BHA and BHT. Protein hydrolysated with pepsin also showed significant antioxidant properties compared to positive control (Figure 1C,D). However, hydrolysate with trypsin fraction E4-T exhibited the highest DPPH radical scavenging activity (85.7 ± 1.8%), and iron chelating activity (94.4 ± 1.3%). Herein, fraction E4-T was subjected to RP-HPLC for further purification.

#### 3.2.2. Isolation of Peptide by Reversed-Phase High Performance Liquid Chromatography (RT-HPLC)

**Figure 2:**
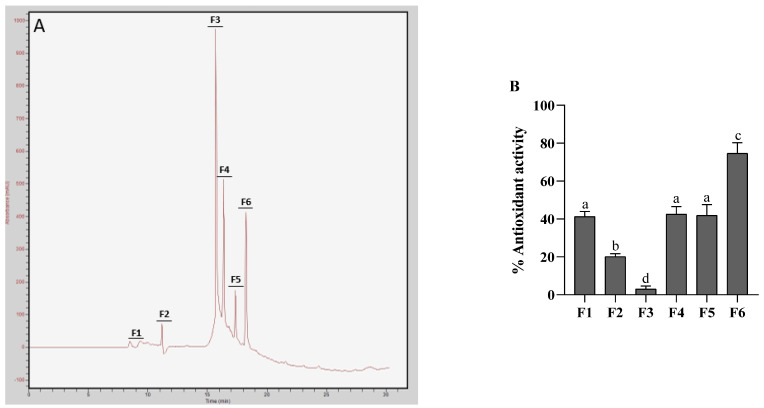
Purification of hydrolysate from trypsin fraction E4-T (MW < 3 kDa) by RP-HPLC on a Thermo C18 column (**A**), and chemical antioxidant activities of corresponding subfractions (**B**). Values were expressed as mean ± SD from triplicate experiments. Bars with different letters in the same group indicated statistical differences (*p* < 0.05, Duncan’s test).

RP-HPLC is the dominant method for the purification of peptides and small protein [25,26,27,28,29,30]. The hydrolysate from trypsin fraction E4-T (MW < 3 kDa), which showed the highest antioxidant activity, were chromatographically fractionated by RP-HPLC on a Thermo C18 column. Hydrophobic peptides from column eluted using an acetonitrile gradient. E4-T was separated into 6 peaks, namely, F1 (RT = 8.5 min), F2 (RT = 11.1 min), F3 (RT = 15.6 min), F4 (RT = 16.3 min), F5 (RT = 17.3 min), and F6 (RT = 18.2 min), as shown in Figure 2A. Antioxidant activities on DPPH of the separated fractions were measured (Figure 2B) and the fraction F6 exhibited the highest DPPH scavenging activity (74.7%). Therefore, F6 was subjected to LC-MS/MS for peptide sequencing.

#### 3.2.3. Identification of Antioxidant Peptide by Liquid Chromatography with Tandem Mass Spectrometry (LC-MS/MS)

**Figure 3:**
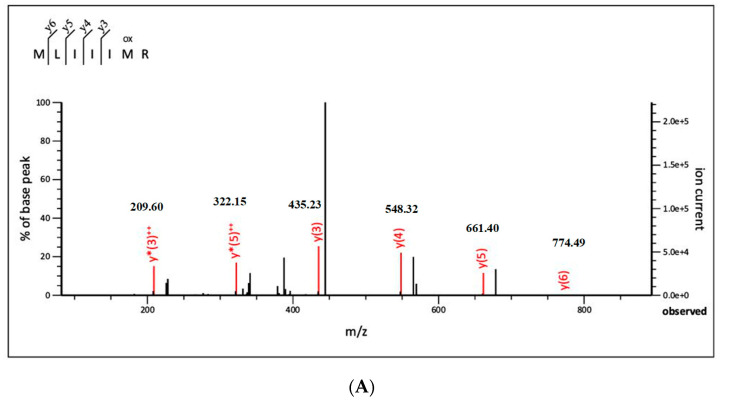

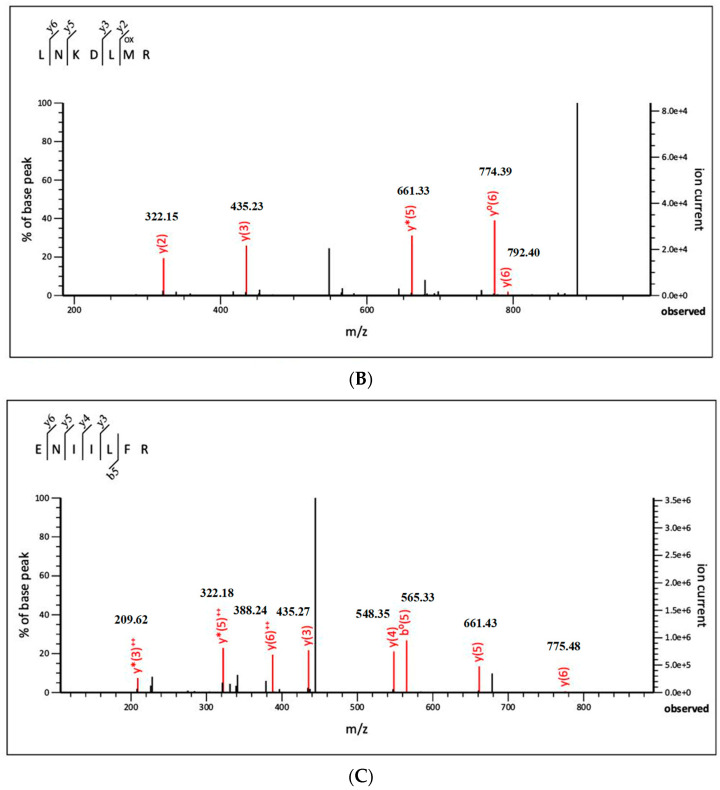
The second mass spectrogram of the identified peptides. (**A**) Mass spectrum analysis of the antioxidant peptide Met-Ley-Ile-Ile-Ile-Met-Arg. (**B**) Mass spectrum analysis of the antioxidant peptide Leu-Asn-Lys-Asp-Leu-Met-Arg. (**C**) Mass spectrum analysis of the antioxidant peptide Glu-Asn-Ile-Ile-Leu-Phe-Arg.

LC-MS/MS is an effective technique because it has been widely applied for analyzing various compounds from biological samples [31,32,33,34]. In this study, the fraction F6 with highest DPPH scavenging capacity was subjected to LC-MS/MS and the results were analyzed by a combination of Mascot searching and MassLynx V4.1 software. Three major novel peptides were separated and identified, and their amino acid sequences were found to be Met-Ley-Ile-Ile-Ile-Met-Arg (MW = 904 Da), Leu-Asn-Lys-Asp-Leu-Met-Arg (MW = 905 Da), and Glu-Asn-Ile-Ile-Leu-Phe-Arg (MW = 903 Da). MS/MS spectra of each identified peptide are shown in Figure 3.

## 4. Discussion

Natural antioxidant peptides have been widely studied including soybean [35], mushroom (*Ganoderma lucidum*) [36], oyster (*Saccostrea cucullata*) [37], scalloped hammerhead (Sphyrna lewini) cartilage [38], and *Bacillus* strain CBS73 [39]. The bioactive peptides, derived from proteins of silkworm pupae (*Bombyx mori*), have been applied in a number of medical applications, such as immunomodulatory, antimicrobial, anticancer, and antioxidant activity [40,41,42,43,44], but proteins derived from *S. ricini* have not attracted much attention.

The bioactive peptide can be generated by hydrolysate capability, which depends on processing conditions, type of enzymes, and molecular weight of the isolated peptides [45,46]. The most common enzymes used for the synthesis of protein hydrolysate are alcalase, neutrase, chymotrypsin, pepsin, and trypsin [47,48,49,50]. In this study, we used trypsin and pepsin to generate protein hydrolysates. Trypsin is a family of serine endopeptidases, with preferential cleavage at carboxyl group of Arg-|-Xaa, and Lys-|-Xaa, produced in the pancreas of many vertebrates [25]. Our study found that the hydrolyzed protein by trypsin with a molecular weight lower than 3 kDa exhibited the highest antioxidant activity. This is in agreement with various reports which indicated that the length of peptide affects bioavailability. Short chains show the better bioactivity than long chains [45,46,47,48,49,50].

Reversed-phase high performance liquid chromatography (HPLC) has become the method of choice for the purification of peptides and small proteins from natural sources [51]. Purification of protein hydrolysate from trypsin fraction E4-T by RT-HPLC was divided into six peaks. Fraction 6 (F6) with the highest antioxidant activity is the last fraction, standing for highly hydrophobic property. This fraction was subjected to LC-MS/MS. Three major novel peptides were separated and identified, and their amino acid sequences were found to be Met-Ley-Ile-Ile-Ile-Met-Arg (MW = 904 Da), Leu-Asn-Lys-Asp-Leu-Met-Arg (MW = 905 Da), and Glu-Asn-Ile-Ile-Leu-Phe-Arg (MW = 903 Da). The results of this study are consistent with the findings of the previous research that low molecular weight peptides exert a significant effect on the antioxidant activities of peptides. Among them, the composition and the sequence of amino acids have the impact on the antioxidant activity [45,46,47,48,49,50].

Previous studies have found that hydrophobic amino acids have been critical to the antioxidant activities of peptides, such as Gly-Phe-Thr-Gly-Pro-Pro-Gly-Phe-Asn-Gly (MW = 950 Da) from scalloped hammerhead (*Sphyrna lewini*) cartilage [38], Leu-Ala-Asn-Ala-Lys (MW = 515 Da) from oyster (*Saccostrea cucullata*) [37], and Phe-Lys-Gly-Pro-Ala-Cys-Ala (MW = 692 Da) from *Bombyx Mori* [52]. A high proportion of hydrophobic amino acids has been reported in peptides with high antioxidant activity, compared to other hydrophilic amino acids [53]. As indicated, the antioxidant properties of the peptides were closely associated with their molecular weight, amino acid composition and sequence, and molecular structure [54]. Here, the identified peptides fell within a molecular weight range of 903–905 Da, consistent with previous results showing that antioxidant peptides typically contained 2–10 amino acid residues [34,35,36,37,38,39]. Moreover, it was evidenced that hydrophobic and antioxidant amino acids, such as Ile (I), Leu (L), Met (M), Phe (F), Tyr (Y), and Val (V) significantly contributed to the radical scavenging activities of peptides by enhancing their interactions with lipids or acting as potent proton/hydrogen donors [55,56,57,58].

## 5. Conclusions

In conclusion, this study suggests that antioxidative peptides obtained from *S. ricini* pupa protein, extracted by hydrolysate methods with trypsin, has significantly different antioxidant activity compared to synthetic antioxidants BHT and BHA. Protein hydrolysate from *S. ricini* pupa could be another important peptide that plays a key role in antioxidant activity. The benefits of antioxidants and their application can be instructive for the food industry to reduce the side effects resulting from the use of synthetic antioxidants.

## Figures and Tables

**Table 1:** DPPH radical scavenging activity of protein hydrolysates from *S. ricini* pupae.

Samples	DPPH Radical Scavenging Activity (IC_50_ μg/mL)
Crude protein	1808.1 ± 98.0 ^b^
Protein hydrolysate with trypsin	1406.6 ± 86.9 ^a^
Protein hydrolysate with pepsin	1495.6 ± 101.7 ^a^
BHA	1304.5 ± 75.4 ^a^

Values are mean of three replicate determinations (n = 3) ± standard deviation. Mean values followed by different superscripts (^a,b^) in a column are significantly different (*p* < 0.05; ANOVA, followed by Tukey’s multiple comparison test).

**Table 2:** Iron chelating activity of protein hydrolysates from *S. ricini* pupae.

Samples	Iron Chelating Activity (IC_50_ μg/mL)
Crude protein	300.7 ± 38.2 ^a^
Protein hydrolysate with trypsin	286.5 ± 48.7 ^a^
Protein hydrolysate with pepsin	189.3 ± 57.1 ^b^
BHA	297.8 ± 29.6 ^a^

Values are mean of three replicate determinations (n = 3) ± standard deviation. Mean values followed by different superscripts in a column (^a,b^) are significantly different (*p* < 0.05; ANOVA, followed by Tukey’s multiple comparison test).

## Data Availability

Not applicable.

## Sample Availability

Samples of the *S. ricini* pupae are available from the authors.
